# Clinical characteristics and surgical outcomes following cardiac myxoma resection

**DOI:** 10.34172/jcvtr.025.33237

**Published:** 2025-03-18

**Authors:** Nootan Hadiya, Madhur Kumar, Rimy Parshad, Poorna Chandar, Anubhav Gupta

**Affiliations:** Department of Cardiothoracic & Vascular Surgery Vardhman Mahavir Medical College & Safdarjung Hospital, New Delhi, India

**Keywords:** Cardiac myxoma, Outcome, Survival, Recurrence

## Abstract

**Introduction:**

Cardiac myxomas are the most common primary cardiac neoplasm (30-50%) with clinical incident of 0.5/ million population. Tranthoracic echocardiography remains the investigation of choice. Surgical excision is curative. The present study aims to analyze demographic and clinical characteristics as well as surgical outcomes in terms of mortality and recurrence of cardiac myxoma.

**Methods:**

Thirty patients of cardiac myxoma who met the inclusion criteria during study period study period, January-2018 to April-2024 were included. Data was analyzed for demographic characteristics, echocardiographic findings of myxoma and associated valve lesion, associated valve surgery and survival outcome.

**Results:**

Of all subjects, 83.33% presented with dyspnea. Majority of myxoma, 76.67% were attached to interatrial septum. Overall survival at 1- and 3- year was 91.23%. Recurrence free survival at 1-, 3- years and end of this study were 100%, 84.71% and 84.71% respectively. Myxomas with valvular incompetence are rare entity and there is paucity of data and evidences recommending concomitant valve intervention in such cases. There were no immediate peri-operative deaths, however, in contrast to other studies; surgical site infection was the most common post operative complication. Overall survival at 1- and 3- year was 91.23%. Recurrence free survival at 1-, 3- years and end of this study were 100%, 84.71% and 84.71% respectively. Recurrence occurred in first- and third-year following surgery.

**Conclusion:**

Study highlights decent outcomes following cardiac myxoma resection. Case specific concomitant valve intervention spiral the success of surgery.

## Introduction

 Primary cardiac tumours are rare with a frequency of about 0.02%.^1^

 Cardiac myxoma are neoplasm of multipotent mesenchymal cells in subendocardial tissue^2^, are most common primary cardiac neoplasm (30-50%) with clinical incident of 0.5/ million population.^3^ Myxoma are particularly frequent from 4^th^ - 6^th^ decade of life with female preponderance (2:1).^4-6^ Majority of myxoma are found in left atrium (75%) followed by right atrium (20%) & rarely (3-4%) in ventricles.^7-11^ More than 90% are sporadic & solitary, 20% occurs in familial clusters as a part of carney complex.^2^ The clinical manifestation of cardiac myxoma occurs either as hemodynamic consequences, systemic or pulmonary embolism and systemic or constitutional symptoms.^5,6,12^ Transthoracic echocardiography remains the investigation of choice.^3,13^ Surgical excision is curative.^4,12^ Recurrence occurs due to tumour implantation, incomplete removal & growth from new focus.^7,14^

 The aim of this study is to analyse demographic and clinical characteristics as well as surgical outcomes in terms of mortality and recurrence of cardiac myxoma.

## Materials and Methods

 The retrospective study was conducted in the department of cardiothoracic & vascular surgery, VMMC & Safdarjung hospital, New Delhi. The data of all patients who had undergone surgery with pathologically confirmed cardiac myxoma during study period, January-2018 to April-2024 were retrieved from medical records department. The mean follow up period was 2.41 ± 2.04 years.

 The data was analysed for demographic characteristics, echocardiographic findings of myxoma and associated valve lesion, associated valve surgery and survival outcome.

 All patients underwent transthoracic echocardiography (TTE) specifically for site of attachment of myxoma and associated valve lesion. Preoperative Coronary angiography was routinely performed in all patients over 40 year of age irrespective of gender. Clinical & Echocardiography parameters were assessed during long term follow- up.

###  Surgical technique

 All procedures were conducted via median sternotomy, with systemic heparinization and total cardiopulmonary bypass with standard aorto-bicaval cannulation and antegrade cold cardioplegic arrest. Cavae snugged. In all patients myxoma was excised via right atriotomy with 5-mm free margin of interatrial septum subsequently sent for histopathology. On the basis of preoperative transthoracic echocardiogram with aid of on-table transesophageal echocardiogram, patients with moderate mitral regurgitation and/or moderate tricuspid regurgitation with tricuspid index > 21mm/m^2^ simultaneously underwent surgical correction.

 Mitral valve repair/replacement was performed through transseptal approach following excision of myxoma. Interatrial septum was reconstructed with a patch. Rewarming was started. Right atrium was closed. Cavae desnugged. Aortic cross-clamp was removed. Root vent on. Patient was gradually weaned off cardiopulmonary bypass.

###  Statistical Analysis

 The presentation of the Categorical variables was done in the form of number & percentage (%). On the other hand, the quantitative data was presented as the means ± SD & as median with 25^th^ & 75^th^ percentiles (interquartile range). Kaplan meier survival analysis was done in the Microsoft EXCEL spreadsheet & final analysis was done with the use of statistical package for Social Sciences (SPSS) software, IBM manufacturer, Chicago, USA, version 25.0.

## Results

 We studied 30 patients of pathologically confirmed cardiac myxoma with demographic characteristics as shown in Table 1. During preoperative assessment, it was found that majority of the patients presented with normal sinus rhythm 26 (86.67%), atrial fibrillation with control ventricular rate was present in 4 (13.33%).

**Table 1:** Demographic characteristics distribution

**Demographic Characteristics**	**Frequency**	**Percentage**
Age (years)		
< = 20 years	2	6.67%
20 - 40 years	10	33.33%
40 - 60 years	13	43.33%
> 60 years	5	16.67%
Mean + - SD	44.1 ± 16.3	
Median (25^th^-75th percentile)	46.5 (34.25- 57.25)	
Range	72	
Gender		
Female	18	60.00%
Male	12	40.00%

 Most common presenting symptom was dyspnea 25 (83.33%) followed by palpitation 18(60%) & chest pain 10 (33.33%). History of cerebrovascular accident was elicited in 5 (16%) patients. Transthoracic Echocardiography (TTE) was performed in all patients. Coronary angiography was performed in 12 (40%) patients of which 2 (6.67%) patients showed single vessel disease, 1 (3.33%) showed non critical left anterior descending artery plaque. Associated valve abnormalities were present in 13 (43.33%) patients (Table 2).

**Table 2:** Associated valve lesion

**Associated Valve abnormalities**	**Frequency**	**Percentage**
No Abnormality	17	56.67%
Mild Mitral regurgitation	2	6.67%
Mild Tricuspid regurgitation	3	10.00%
Mild Mitral regurgitation, Mild Tricuspid regurgitation	2	6.67%
Moderate Mitral regurgitation	2	6.67%
Moderate Tricuspid regurgitation	3	10.00%

**Figure 1:**
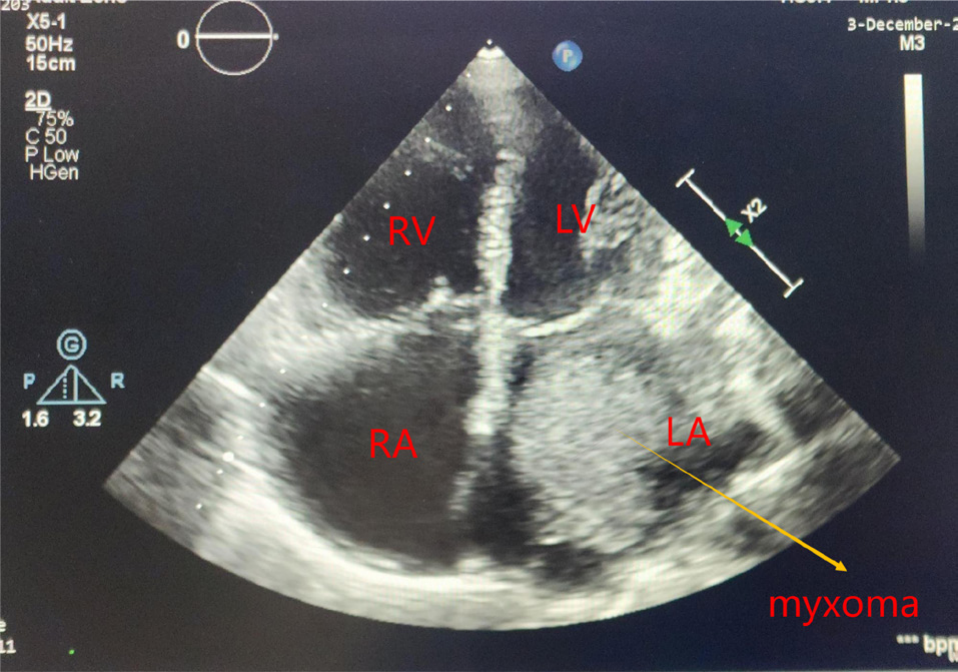
Echocardiography image of left atrial myxoma

**Figure 2:**
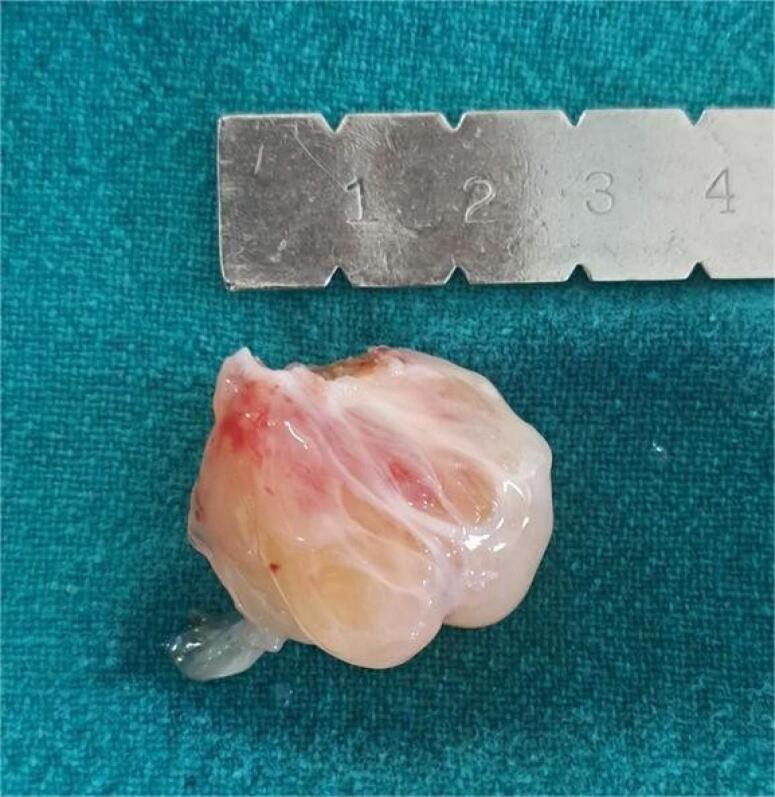
Gross specimen of myxoma

**Figure 3:**
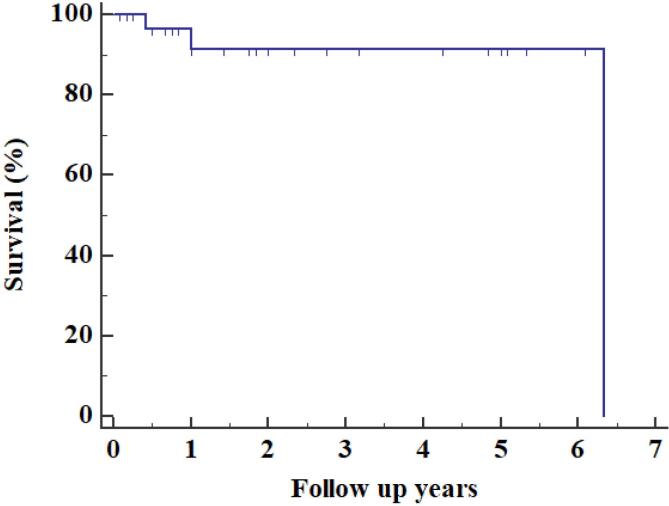
Kaplan- Meier survival analysis curve to assess overall survival

**Figure 4:**
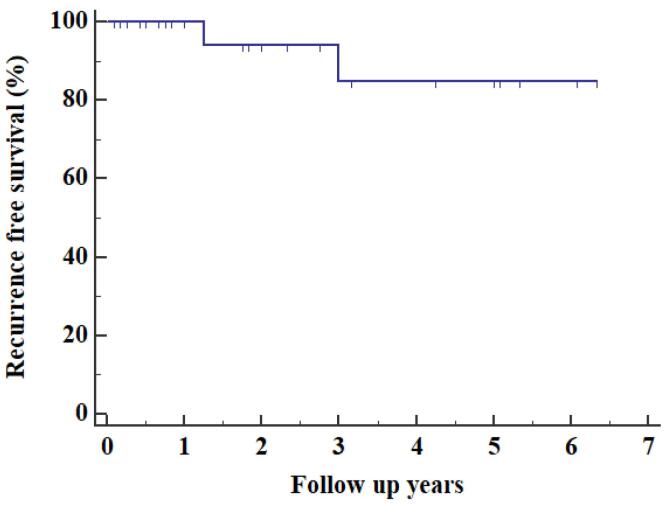
Kaplan-Meier survival analysis curve to assess recurrence free survival

 Additional imaging required in the form of cardiac computed tomography (CT) 3 (10%), Contrast enhanced computed tomography (CECT) of thorax 1(3.33%) & cardiac magnetic resonance imaging (MRI) 1 (3.33%), either due to suboptimal quality of TTE or atypical location of the myxoma. MRI brain 1(3.33%) & NCCT Head 1 (3.33%) was performed in patients with history of ischemic stroke. Most common site of myxoma was left atrium 25 (83.33%) as shown in Figure 1, followed by right atrium 4 (13.33%) & right ventricle outflow tract 1(3.33%) with mean size of 5.17 ± 1.46 cm. Most of them were attached to interatrial septum 23 (76.67%). Gross appearance of myxoma shown in Figure 2. Mean preoperative ejection fraction was 55.57 ± 8.59%. Associated valve lesions are shown in Table 2. Concomitant valve surgery performed shown in Table 3. Mitral valve surgery was most commonly performed. Among Mitral valve surgery, 3(10%) were due to Rheumatic valvular disease & 1(3.33%) due to degenerative valvular disease. Remaining Mitral valve surgery 2 (6.67%) due to myxomatous involvement of valve & all 5 (16.67%) Tricuspid valve repairs were done for annular dilatation. Post-operative complications occurred in 6 (20%) patients, most common being superficial surgical site infection 3(10%), followed by atrial fibrillation without conduction disturbance 1(3.33%) & pleural effusion 1(3.33%). Mean duration of intensive care unit (ICU) stay was 4 ± 1.84 days. Mean duration of hospital stay was 16.53 ± 6.57 days. Only one patient (3.33%) died during ICU stay due to low cardiac output syndrome. During follow up, mortality was noted in 2 (6.90%) due to other reasons, one in 1^st^ year & other in 2^nd^ year. Recurrence was observed in 2 (6.67%) during follow up, one after 1- year & one after 3-years of surgery. Long term cumulative and recurrence free survival was analyzed using Kaplan- Meier method as shown in Figure 3 & Figure 4 respectively.

**Table 3:** Associated valve surgery

**Additional valve repair/ replacement required**	**Frequency**	**Percentage**
Not required	19	63.33%
Mitral valve repair	5	16.67%
Tricuspid valve repair	4	13.33%
Mitral valve repair + Tricuspid valve repair	1	3.33%
Mitral valve replacement	1	3.33%

## Discussion

 In our study, cardiac myxoma were more common in female (60%), as comparable with Velu et. al.^15^, Lee et al^16^ & Nektaria et al^17^ The mean age of our study population was 44.1 ± 16.3 years, as opposed to a study from South Korea^16^ and Argentina.^18^ Our findings were similar to a study of 49 patients at Karl Franzens University, Austria which showed female: male – 3:1.^19^ Dyspnea was the most common symptom (83.33%) as also seen in Goswami et al^20^ & Pinede et al^l6^ evidenced by a high percentage of patients with signs of clinical heart failure.

 We noticed thromboembolic events in 16% of patients which was similar to Kacar et al^4^ Constitutional symtoms occurred in 8% patients which was lesser compared to other studies.^6,21^ Transthoracic Echocardiogram is the investigation of choice with 90-96% of accuracy in diagnosing cardiac myxoma.^4,5,12^ In patients with poor acoustic shadow & atypical presentation, multimodality imaging like CT or Cardiac MRI is recommended which provides additional benefit in terms of tissue characteristics & its topographic relations.^4,22,23^ In our study, left atrium was the most common site (83.33 %) with interatrial septum being most common implantation site (76.67%), as also noted in cianciulli et al^18^ & other local^24^ & international studies.^6,25^ Surgical resection is the only treatment for any myxoma & surgical approach depends on the site & attachment of mass.^7^ In this study, concomitant mitral valve repair 5 (16.67%) and replacement 1 (3.33%) was performed. Myxomas with valvular incompetence are rare entity and there is paucity of data and evidences recommending concommitent valve intervention in such cases.^26^ In the landmark study of Lee et al^16^ in their 30-year experience of surgical exicision of cardiac myxoma, 5 (5.3%) underwent concurrent mitral valve intervention in the form of performed mitral valve repair in two patients and replacement in other three patients. Similarly, Potey et al had also performed mitral valve intervention along with myxoma resection.^27^

 There were no immediate peri-operative deaths, however, in contrast to other studies, surgical site infection was the most common post operative complication.^4,5^ 30- day mortality rate after myxoma excision was 0-10%.^28^ Our study had mortality rate of 10%, comparable to Nektaria et al.^17^ Overall survival at 1- and 3- year was 91.23%. Recurrence free survival at 1-, 3- years and end of this study were 100%, 84.71% and 84.71% respectively. Recurrence occurred in first and third year following surgery.

 The recurrence rate following myxoma resection was reported to be less than 10%.^28^ Recurrence is relatively common in familial cases, rare in sporadic cases, mainly occurs in first three to four years.^29^

 This study was retrospective cohort study, however this patients should be operated as early as possible due to chances of embolism, it is difficult to do a prospective study for this patients. We included a small sample size but still it is comparable & higher than some previously published studies which shows its paucity of occurrence which now increasingly diagnosed because of development of good diagnostics.

## Conclusion

 Clinical outcomes following cardiac myxoma resection are acceptable well supported by concomitant case specific valve intervention. Thorough pre-operative and intra-operative assessment of valve following myxoma resection is imperative. Although recurrence is low, wide resection is recommended.

## Competing Interests

 None.

## Ethical Approval

 Consent was obtained by all participants in this study. Vardhaman Mahavir Medical College & Safdarjung Hospital issued approval.
